# The Role of Visual Noise in Influencing Mental Load and Fatigue in a Steady-State Motion Visual Evoked Potential-Based Brain-Computer Interface

**DOI:** 10.3390/s17081873

**Published:** 2017-08-14

**Authors:** Jun Xie, Guanghua Xu, Ailing Luo, Min Li, Sicong Zhang, Chengcheng Han, Wenqiang Yan

**Affiliations:** 1 School of Mechanical Engineering, Xi’an Jiaotong University, Xi’an 710049, China; luoailing@xjtu.edu.cn (A.L.); min.li@xjtu.edu.cn (M.L.); zhsicong@xjtu.edu.cn (S.Z.); handeno.2@stu.xjtu.edu.cn (C.H.); a3115001076@stu.xjtu.edu.cn (W.Y.); 2 State Key Laboratory for Manufacturing Systems Engineering, Xi’an Jiaotong University, Xi’an 710049, China

**Keywords:** brain-computer interface, steady-state visual evoked potential (SSVEP), steady-state motion visual evoked potential (SSMVEP), visual noise, mental load, fatigue

## Abstract

As a spatial selective attention-based brain-computer interface (BCI) paradigm, steady-state visual evoked potential (SSVEP) BCI has the advantages of high information transfer rate, high tolerance to artifacts, and robust performance across users. However, its benefits come at the cost of mental load and fatigue occurring in the concentration on the visual stimuli. Noise, as a ubiquitous random perturbation with the power of randomness, may be exploited by the human visual system to enhance higher-level brain functions. In this study, a novel steady-state motion visual evoked potential (SSMVEP, i.e., one kind of SSVEP)-based BCI paradigm with spatiotemporal visual noise was used to investigate the influence of noise on the compensation of mental load and fatigue deterioration during prolonged attention tasks. Changes in *α*, *θ*, *θ* + *α* powers, *θ*/*α* ratio, and electroencephalography (EEG) properties of amplitude, signal-to-noise ratio (SNR), and online accuracy, were used to evaluate mental load and fatigue. We showed that presenting a moderate visual noise to participants could reliably alleviate the mental load and fatigue during online operation of visual BCI that places demands on the attentional processes. This demonstrated that noise could provide a superior solution to the implementation of visual attention controlling-based BCI applications.

## 1. Introduction

Brain-computer interfaces (BCIs) traditionally harness intentionally-generated brain signals to control devices that can, in turn, be potentially helpful for disabled individuals by replacing the usual channels of communication and control [1]. A variety of methods for monitoring brain activities might serve as a BCI. In addition to electroencephalography (EEG), these include magnetoencephalography (MEG) [2], functional magnetic resonance imaging (fMRI) [3], functional near-infrared spectroscopy (fNIRS) [4,5,6], and more invasive electrophysiological methods. Among them, EEG and related methods have high time resolution, lower environmental limits, require relatively inexpensive equipment [7], and have been largely used in practical BCI applications. Generally, two types of EEG patterns of the P300 component of the event-related potential (ERP) [8,9] and steady-state visual evoked potential (SSVEP) are more practically used to develop visual BCI systems because they support large numbers of output commands, and need little training time [10]. The P300-based BCI has relatively robust performance for target detection. Although its information transfer rate (ITR) is at a medium level, unlike an SSVEP-based BCI, it does not cause some participants to feel annoyed or fatigued by the flickering stimuli [11]. On the other hand, due to the advantages of high-level ITR, high tolerance to artifacts, and robust performance across users, the SSVEP-based paradigm has been widely used in BCI applications. As a visual spatial selective attention-based BCI paradigm, SSVEP BCI requires users to concentrate on the visual stimulus to generate sufficiently strong responses. However, due to high brightness, overstimulation, and repetitive attentional demands, SSVEP BCI may easily result in a high mental load and users may become fatigued. In the present context, mental load can be defined as a measure of the amount of mental resources engaged in a task. The mental load level is considered as an index of task difficulty [12]. In addition, the visual or mental fatigue, which is partially induced by cognitive load [13,14], is associated with tiredness or exhaustion and results in a decrease in cortical arousal and BCI performance [15]. When people experience high mental load and become fatigued in SSVEP-BCI tasks, they might be easily disturbed by distracting stimulations, which distract their attention from targets due to the competition for attentional resources [16]. Therefore, mental load and visual fatigue should be considered when designing spatial selective attention-based SSVEP BCIs.

To assess mental load and fatigue, EEGs in *α* band (~8–13 Hz) and *θ* band (~4–7 Hz) can be used to distinguish different levels of mental states. The occurrence of *θ* activity is associated with drowsiness, attention, and processing of cognitive and perceptual information. The *α* waves appear during relaxed conditions, at decreased attention levels, and in a drowsy, but wakeful, state. An overall decrease in *α* power has been linked to increased alertness and task load, in general [17,18,19]. Global increases in attentional demands and corresponding mental load are most associated with a decrease in *α* power and an increase in *θ* activity [20,21]. Furthermore, changes in *α* and *θ* powers seem to be the most robust objective indicators of not only mental load but also fatigue. Under decreased attention and arousal levels, there are progressive increases in *α* and *θ* activities in resting spontaneous EEGs [22,23,24], this probably reflecting a decrease in cortical activation and task performance [25,26]. Therefore, *α* and *θ* activities can be adopted to evaluate the degree of mental load and fatigue in the context of BCI applications.

Noise is a ubiquitous random perturbation commonly found in neural systems of humans and other mammals [27]. Noise is typically considered as detrimental to cognitive performance. However, recent studies were able to demonstrate that, somewhat counter-intuitively, irrelevant noise exposure can be beneficial for performance in cognitive tasks [28]. This phenomenon is labeled as stochastic resonance (SR), or stochastic facilitation, in a broader sense [29,30], which was introduced in the early 1980s by Benzi [31], describes the phenomenon whereby random fluctuations or noise can enhance the detectability and/or synchronization of a weak signal in certain non-linear dynamic systems, i.e., noise paradoxically does not worsen, but improves, system capability, and can be used to account for noise-induced improvement in cognitive performance [32]. The “beneficial” effects of noise in both experimental studies and theoretical investigations of neural systems have shown particular circumstances in which synchronization of neuronal firing was enhanced by the presence of random fluctuations [33,34]. A moderate level of noise is beneficial for achieving perception, cognition, or action tasks [35]. This is due to nerve cells in sensory organs being described as a thresholding system, so the neuronal membrane voltage which is not large enough to cross the intrinsic threshold non-linearity alone would be properly assisted with noise at a moderate intensity to accomplish threshold-crossings. Therefore, the appearance of SR can be roughly explained by that addition of noise that effectively turns neurons from sub- to supra-threshold. However, too little noise does not add the power required to bring the signal over the threshold, whereas too much noise overpowers the signal, leading to deterioration in attention and performance [27,36].

Given that noise has been shown to facilitate sensory processing in visual BCI applications [37], its influence on neural processing would rather permeate every level of the nervous system and should, likewise, be relevant to the implementation of higher cognitive functions, such as arousal and attention [38]. In this work, we proposed the use of a novel steady-state motion visual evoked potential (SSMVEP, i.e., one kind of SSVEP)-based online BCI paradigm associated with spatiotemporal visual noise to investigate the influence of stochastic facilitation on the capacity of mental load and fatigue experienced during prolonged attention tasks. To benchmark the effect of mental load and fatigue occurring in non-noise and noise-tagged stimulation procedures, we evaluated changes in SSMVEP amplitudes and signal-to-noise ratios (SNRs), spectral indices in the *α* and *θ* bands, as well as online accuracy and correct response time, which characterize BCI accuracy and efficiency.

## 2. Materials and Methods 

### 2.1. Participants and Recordings

Twelve graduate students (seven males and five females) from Xi’an Jiaotong University (Shaanxi, China), aged between 23 and 29 years old, participated in this study. All participants had normal or corrected-to-normal vision and had experienced SSVEP BCIs before. However, they were new as to the visual noise-masked SSMVEP-based BCI paradigm. They had no history of psychiatric or neurological disorders and no visual perception disturbances or impairments were reported. Before starting the experiment, all participants gave their informed written consent in compliance with the guidelines approved by the institutional review board of Xi’an Jiaotong University.

EEG signals were recorded from the occipital head (Oz) using a g.USBamp system (g.tec Medical Engineering GmbH, Schiedlberg, Austria) at a sampling rate of 1200 Hz in order to ensure that trials encompassed single cycles of three stimulation frequencies exactly. This allowed each stimulation frequency to be fully contained within an individual FFT “bin”, thus alleviating spectral leakage [39,40]. EEG signals were referenced to a unilateral earlobe and grounded at the forehead (Fpz). An online band-pass filter from 2 to 100 Hz and a notch filter between 48–52 Hz were applied to remove artifacts and power line interference.

### 2.2. Stimulation Designs

Motion-reversal visual stimulations were introduced into the spatial selective attention-based steady-state BCI paradigm. Here the ‘‘steady-state’’ brain responses were evoked by mirror movements which oscillated in two opposite directions. For the presentation of such oscillating motion, each of the two motion directions would be presented at half cycle time and then be replaced by the other direction motion, comprising one stimulus period. The direction change rate served as the stimulation frequency. Its first subharmonic frequency equaled the sinusoid frequency [41].

**Figure 1:**
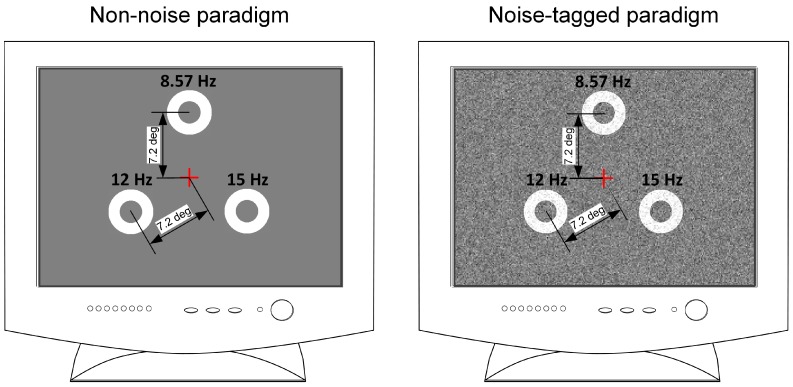
Distribution of three noise masked targets on the computer screen with noise standard deviations of 0 and 40. The cross indicating the center of the monitor was not presented on the screen. The eccentricity from the center of the monitor to that of each target was in a visual angle of 7.2°.

In this study, three motion-reversal targets were simultaneously presented to participants through a gamma-corrected 22-inch Dell LCD monitor at a resolution of 1024 × 768 pixels. Each participant was situated 70 cm from the screen with the center at eye level. Three targets were uniformly arranged in an equilateral triangle. The eccentricity from the center of the monitor to that of each target was in a visual angle of 7.2°. Each target was created using a motion ring object whose width was kept constant as half the radius of the circular region (Michelson contrast of 98.8%) throughout the motion reversal procedure. The circular area was 4.8° in diameter, in accordance with previous studies showing that a stimulus size beyond 3.8° would saturate VEP responses [42]. The phase of the motion ring was temporally sinusoidally shifted so as to produce the motion reversal procedure, which included the inward contraction and outward expansion motions alternately. Here the contraction of the motion ring was implemented by its phase shift from 0 to π, and then expansion motion was achieved with phase shift from π back to 0. The three targets moved at unique, constant, and mutually-irrational stimulation frequencies. In accordance with the integer division of the 60-Hz refresh rate, motion-reversal frequencies of 15 Hz, 12 Hz, and 8.57 Hz were assigned to the lower right, lower left, and upper targets, respectively. With the same paradigm, but adding a moderate visual noise, a noise-masked visual stimulation was applied as illustrated in Figure 1. In the present study, the spatiotemporal noise referred to as dynamic changes of spatial noise speckles. Each noise speckle subtended a square area of 5 min of visual angle and obeyed Gaussian intensity distributions with a mean gray level of 128 and a standard deviation of 40. The spatiotemporal noise was masked to targets and was updated in 1/60 s. The stimulation design and the motion reversal procedure were scheduled according to our earlier studies [37,43]. Presentation of the stimulation was controlled by the Psychophysics Toolbox (http://psychtoolbox.org/) [44,45].

### 2.3. Online BCI Tasks

**Figure 2:**
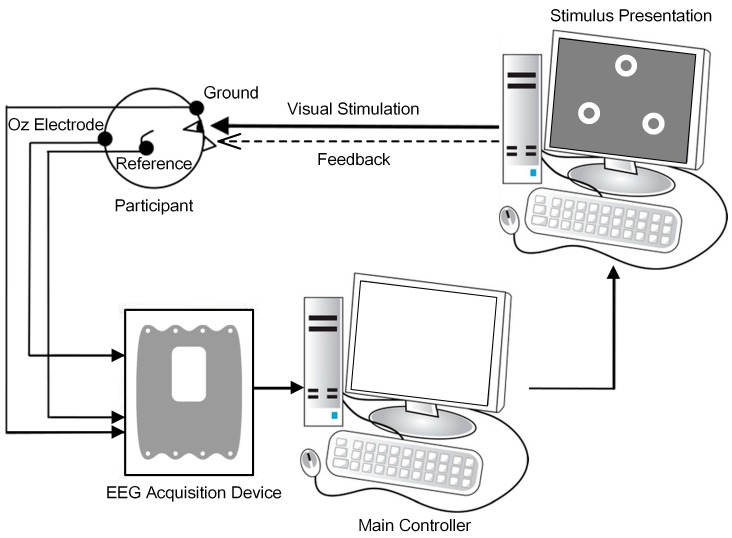
The overall BCI system diagram.

**Figure 3:**
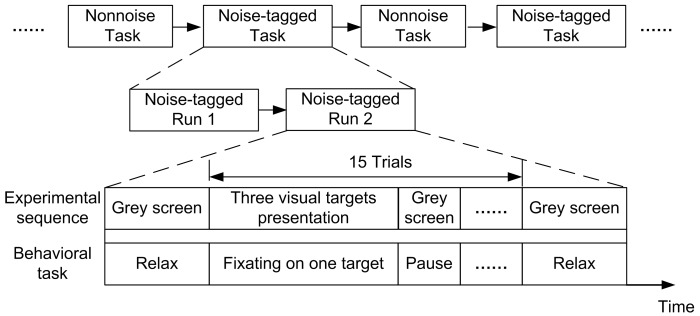
The timing of the experimental sequence and behavioral task. For each participant, the experimental tasks alternated every two runs like “Non-noise run – Non-noise run – Noise-tagged run – Noise-tagged run – Non-noise run – Non-noise run …”.

The overall BCI system diagram was illustrated in Figure 2. The online BCI tasks were categorized into non-noise and noise-tagged tasks. Participants were asked to attend to every 15 Hz, 12 Hz, and 8.57 Hz stimulation sequentially, which constitutes a stimulation sequence. For each participant, each task contained 4–8 runs and each run consisted of five stimulation sequences with 15 trials. The experimental tasks alternated every two runs like “Non-noise run – Non-noise run – Noise-tagged run – Noise-tagged run – Non-noise run – Non-noise run …”, as illustrated in Figure 3. The online BCI tasks were implemented in a semi-synchronous way wherein the duration of stimulation varied from 2 to 10 s in steps of 0.5 s, with a fixed 5 s inter-trial interval (ITI). In every trial, a one-second red cue displayed above a specific target and instructed participants to pay attention to that target. The duration of stimulation increased until the target was identified twice as being the same target in succession (either correct or not). Once the target was identified, a one-second green cue appeared in the center of the screen to mark the result and this trial ended. If brain responses failed to meet the detection criteria beyond 10 s for any of the three targets, this trial would end with no cue. Resting spontaneous EEGs were collected at ITIs. Participants were not allowed to blink eyes or move their bodies during each run and they were asked to fixate on the center of screen during the ITI periods. Therefore, the horizontal or vertical electrooculogram (EOG) signals were not recorded and trials contaminated by few artifacts were also not excluded.

### 2.4. Online Target Identification

For each trial, a *GT^2^_circ_* test [46] was used to check the presence of SSMVEP on the statistics of responses at each stimulation frequency. Three rectangular windows involving three cycles of each stimulation frequency, i.e., 480 data points for 15 Hz stimulation, 600 data points for 12 Hz, and 840 data points for 8.57 Hz, were sequentially slid over each trial with one-cycle overlap, i.e., 160 data points for 15 Hz, 200 data points for 12 Hz, and 280 data points for 8.57 Hz. The resulting data segments were submitted to the fast Fourier transform (FFT), creating four-feature vectors with complex Fourier components from each stimulation frequency and its sub-harmonic. The *GT^2^_circ_* test provides a probability to determine whether feature vectors are consistent with random fluctuations alone or if they infer the presence of periodic components beyond a given confidence level. In our study, the confidence level was set at 0.99. The stimulation with the maximal confidence probability exceeding the confidence level would be statistically identified as the attended target.

### 2.5. Statistical Analysis

To investigate the mental load and fatigue effects in both non-noise and noise-tagged BCI tasks, the changes of *α* and *θ* powers and the *θ*/*α* ratio were used in mental load evaluation, and *α*, *θ*, *θ* + *α* powers, SSMVEP properties of amplitude, SNR, and online accuracy were used to evaluate potential fatigue effects. In this study, Fourier powers of *α* and *θ* were quantified by calculating the band power with Welch’s power spectral density estimation in bins of 0.4 Hz. To avoid the overlap of *α* rhythms (~8–13 Hz) with 8.57 Hz stimulation, and to avoid the overlap of *θ* rhythms (~4–7 Hz) with the sub-harmonic of 8.57 Hz (i.e., the 4.28 Hz component), the *α* and *θ* powers were extracted through the frequency band 9–13 Hz and 4.5–7 Hz, respectively. For the convenience of the analysis, the 12 Hz stimulation was not included in the scope of this study due to the fact that this stimulation frequency and its sub-harmonic were both involved in the dominant bands of *α* and *θ* rhythms. The SSMVEP amplitude spectra at stimulation frequencies of 15 Hz and 8.57 Hz, and their respective sub-harmonics, were extracted by FFT. Similarly, the SNRs at stimulation frequencies of 15 Hz and 8.57 Hz and their respective sub-harmonics were computed as the ratio between the Fourier power obtained at the target frequency *f* and the mean power value of its adjacent frequencies *f* ± 0.4 Hz [47,48].

Repeated analysis of variance (ANOVA) procedure with Bonferroni correction was applied for the statistical significance analysis of the indices. The Bonferroni correction was employed by means of adjusting for all pairwise comparisons of dependent variables (e.g., noise mode, SSMVEP strength, and EEG band index). ANOVA with polynomial curve fitting (i.e., least-squares regression) statistics was conducted to evaluate the trend in the association between the experimental order and indices. The objective of this trend analysis was to study the trend of the index means across the experimental order and the separate contributions of linearity and nonlinearity. The latter were evaluated by testing each linear and quadratic coefficient against the null hypothesis that the best fitting straight line has a slope of 0 [49]. The level of significance for the statistical tests was set at *p* < 0.05.

## 3. Results

In the following, we first focused on the mental load. It was hypothesized that in BCI tasks, *α* activity would decrease and *θ* power increase along with the increase in mental load [50]. As a consequence of the prolonged BCI usage, participants were then expected to experience mental fatigue, which would be reflected by lower BCI performance, along with reduced SSMVEP amplitude, SNR, and online accuracy.

### 3.1. Influence of Visual Noise on Mental Load

The mental load analysis was restricted to the grand average of *α* and *θ* powers and the *θ*/*α* ratio changes for all participants. The SSMVEP findings concerning mental load were carried out in within on-task EEGs and between non-task and on-task EEGs. To facilitate the subsequent comparison of mental load between non-noise and noise-tagged BCI tasks, we first validated the hypothesis stating that a lower mental load is associated with an increase in *α* power and a decrease in *θ* power in non-task resting spontaneous EEGs rather than in on-task EEGs during goal-directed cognitive tasks [51].

**Figure 4:**
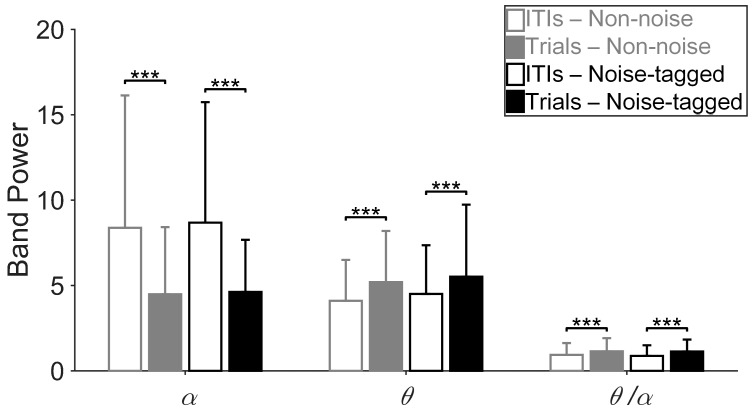
Comparison of mental load indices between non-task ITIs and on-task trials in both non-noise and noise-tagged conditions across participants. The mean values and standard deviation (SD) of *α* and *θ* powers and the *θ*/*α* ratio were calculated across twelve participants. All statistics were assessed by one-way ANOVA, *** *p* < 0.001 between non-task ITIs and on-task trials in both non-noise and noise-tagged BCI applications.

Figure 4 shows the grand-averaged power spectra in *α* and *θ* bands and corresponding *θ*/*α* ratio across twelve participants. The power spectra were measured from non-task and on-task EEGs under both non-noise and noise-tagged stimulation procedures, for which each run consisted of 10 consecutive on-task trials at stimulation frequencies of 15 Hz and 8.57 Hz and 14 non-task inter-trial intervals (ITIs) of 5 s, respectively. To test the above hypothesis in *α* and *θ* powers and corresponding *θ*/*α* ratio, a two-way repeated ANOVA including the factors of noise mode using non-noise vs. noise-tagged conditions on non-task and on-task EEGs was conducted. We found a main effect of non-task ITIs vs. on-task trials (*p* < 0.05 for all comparisons), but no interaction (*p* > 0.05 for all comparisons), implying that for *α* and *θ* powers and corresponding *θ*/*α* ratios, respectively, the relative magnitudes among non-task ITIs vs. on-task trials did not change with the noise mode. Subsequent one-way ANOVA revealed a significant *α* band synchronization in non-task ITIs rather than in on-task trials for both non-noise and noise-tagged BCI applications (*p* < 0.001 for all comparisons). A reversed phenomenon was found significant in *θ* power for both non-noise and noise-tagged conditions (one-way ANOVA: *p* < 0.001 for all comparisons). The corresponding *θ*/*α* ratio revealed significantly lower values during non-task ITIs than during on-task trials across all participants under both non-noise and noise-tagged conditions (one-way ANOVA: *p* < 0.001 for all comparisons). These implied that when performing BCIs, the idle condition, during which participants did not attend any stimulation, would result in apparent lower mental load than the visual attention condition that includes a task.

**Figure 5:**
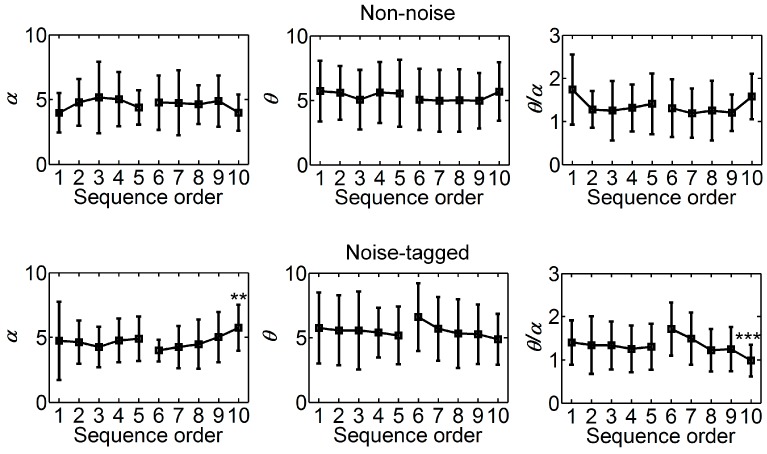
Comparison of mental load indices between on-task stimulation sequences in both non-noise and noise-tagged BCI tasks across participants. The mean values and SD of *α* and *θ* powers and *θ*/*α* ratio were calculated from 10 consecutive stimulation sequences with the same order across twelve participants. All statistics were assessed by one-way ANOVA, ** *p* < 0.01 between five consecutive stimulation sequences in either the first or the second runs of two sequential runs, *** *p* < 0.001 between five consecutive sequences.

Figure 5 shows the mental load indices of *α* and *θ* powers and *θ*/*α* ratio that were calculated from on-task EEGs in both non-noise and noise-tagged BCI tasks across twelve participants. Since participants were asked to attend to every 15 Hz, 12 Hz, and 8.57 Hz stimulation sequentially, which constitutes a stimulation sequence, and also because each run consisted of five stimulation sequences and the experimental tasks alternated every two runs like “Non-noise run – Non-noise run – Noise-tagged run – Noise-tagged run – Non-noise run – Non-noise run …”, the *α* and *θ* powers and *θ*/*α* ratio were summed over the stimulation frequencies of 15 Hz and 8.57 Hz in the 10 consecutive stimulation sequences (i.e., belonging to two sequential runs) with the same order across twelve participants. To evaluate the interaction effect between the noise mode and the stimulation sequence, a two-way repeated ANOVA including the factors of noise mode using non-noise vs. noise-tagged conditions on consecutive stimulation sequences 1–10 was conducted. We found a significant interaction effect between the noise mode and the stimulation sequence in *θ*/*α* ratio (*p* = 0.010), implying that the tendency of *θ*/*α* ratio over 10 consecutive stimulation sequences changed with the noise mode. Here, the overall rising tendency of the *θ*/*α* ratio in the non-noise task and decreased tendency in the noise-tagged task could be noticed in sequence order 6–10 of Figure 5.

Specifically, for the second runs (i.e., sequence order 6–10; Figure 5) of the non-noise BCI task, a trend representing the increase in mental load as the decrease in *α* power and the increase in *θ* power, and in the *θ*/*α* ratio, seemed to be present among the successive sequences, but did not reach statistical significance (*F*(4, 95) = 0.66, *p* = 0.618 for *α* power; *F*(4, 95) = 0.34, *p* = 0.848 for *θ* power; *F*(4, 95) = 1.47, *p* = 0.217 for *θ*/*α* ratio; one-way ANOVA with Bonferroni-corrected post-hoc tests). For the second runs of the noise-tagged task, the reversed phenomena of an overall rise tendency of *α* power could be noticed across successive sequences (ANOVA testing for linear trend: *p* < 0.001), whereas *θ* power and *θ*/*α* ratio decreased progressively with increasing sequence order (*p* = 0.025 for *θ* power; *p* < 0.001 for *θ*/*α* ratio). The sequence differences in *α* power and in *θ*/*α* ratio were also significant (*F*(4, 95) = 3.57, *p* = 0.009 for *α* power; *F*(4, 95) = 5.68, *p* < 0.001 for *θ*/*α* ratio; one-way ANOVA with Bonferroni-corrected post-hoc tests), whereas the difference in *θ* power was not significant (*F*(4, 95) = 1.47, *p* = 0.216). These demonstrated a facilitation of visual noise in alleviating the mental load, as indicated by the increase in *α* power and the decrease in *θ* power and in *θ*/*α* ratio. Additionally, the mental load was worsened in the normal non-noise BCI task.

For the first runs (i.e., sequence order 1–5; Figure 5) of the non-noise BCI task, the findings regarding *α* power and *θ*/*α* ratio presented the characteristics of notably-significant inverted-U- and U-shaped quadratic (i.e., non-monotonic) trends, respectively, as a function of the sequence order. To test the U-shaped relation between the sequence order and the two indices, we entered both linear and quadratic terms in trend analyses. The analysis indicates a U-shaped relation if the quadratic term is significantly different from 0. Here, a positive and statistically significant quadratic term would indicate a U-shaped correlation while a negative and statistically significant quadratic term would indicate an inverted-U-shaped correlation. The polynomial trend analysis on *α* power resulted in a significant negative quadratic term, indicating a significant inverted-U-shaped relationship (ANOVA testing for quadratic trend: *p* = 0.043) against the linear association (insignificant; ANOVA testing for linear trend: *p* = 0.444) between the sequence order and *α* power. This demonstrated that a quadratic trend, in this case an inverted-U-shaped relationship, better fitted *α* power than a linear relationship. Unlike *α* power, *θ*/*α* ratio showed a significant U-shaped relationship associated with the sequence order (ANOVA testing for quadratic trend: *p* = 0.029). For *θ* power, no significant quadratic relationship was noticed (ANOVA testing for quadratic trend: *p* = 0.543). Specifically, in the non-noise BCI task, the magnitude of the grand-averaged *α* power progressively increased and then decreased, while the magnitude of the grand-averaged *θ*/*α* ratio progressively decreased and then increased; both findings illustrate the same phenomenon: mental load first decreased shortly but then increased during the last stimulation sequences of the first runs and until the second runs. In the noise-tagged task, the *α* and *θ* powers and the *θ*/*α* ratio in the first runs behaved with similar tendencies as compared to its respective second runs, but showing an insignificant trend of mental load alleviation for *α* and *θ* powers (ANOVA testing for linear trend: *p* = 0.748 for *α* power; *p* = 0.472 for *θ* power), but a significant trend of mental load alleviation for the *θ*/*α* ratio (ANOVA testing for linear trend: *p* = 0.015). This implied that the very first mental load alleviation in the non-noise task may be derived from its preceding noise-tagged task, and the reason why the mental load alleviation was not so significant in the first runs of the noise-tagged task may result from its preceding non-noise task.

### 3.2. Influence of Visual Noise on Fatigue

**Figure 6:**
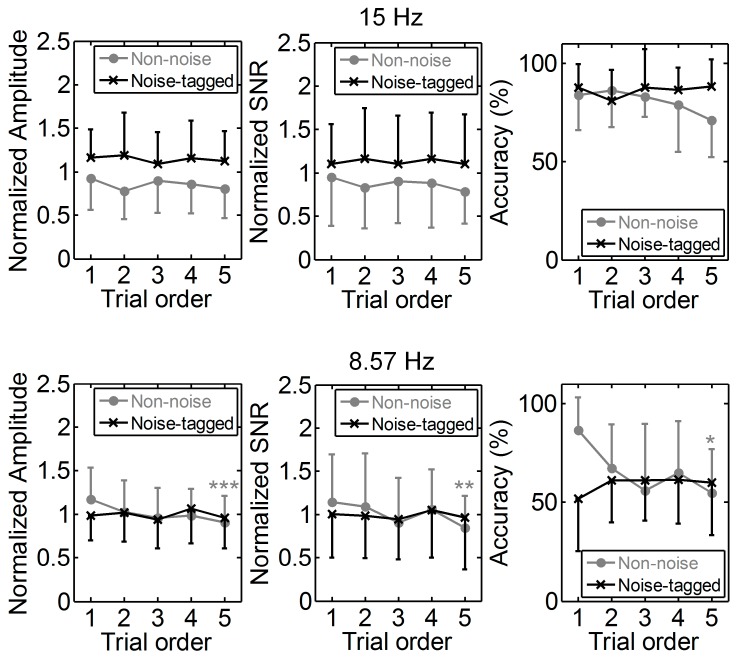
Comparison of fatigue indices of normalized amplitude, SNR, and online accuracy between five consecutive on-task trials in both non-noise and noise-tagged BCI tasks across participants. The grand-averaged mean values and SD of amplitude, SNR, and accuracy were calculated from five consecutive trials of the same order in each run across twelve participants. All statistics were assessed by one-way ANOVA, * *p* < 0.05 between five consecutive trials in both non-noise and noise-tagged tasks, ** *p* < 0.01 between five consecutive trials, *** *p* < 0.001 between five consecutive trials.

Figure 6 indicates the amplitude, SNR, and accuracy differences between different trial orders for both non-noise and non-tagged tasks at different stimulation frequencies across twelve participants. The SSMVEP amplitudes and SNRs at stimulation frequencies of 15 Hz and 8.57 Hz, and their respective sub-harmonics, were extracted from the spectral power of multiple runs of successive trials with the same order. Inter-participant normalization was attained by dividing the amplitude and SNR estimates by the average computed from all amplitude and SNR values of both non-noise and noise-tagged conditions, respectively, but separately for each participant [52]. Three-way repeated ANOVA with Bonferroni correction, which included the factors of “noise mode”, “stimulation frequency”, and “on-task trial”, showed a significant interaction effect between the noise mode and the on-task trial in SSMVEP amplitude and accuracy (*p* = 0.039 for amplitude; *p* > 0.023 for accuracy), implying that the tendencies of SSMVEP amplitude and accuracy over five consecutive on-task trials changed with the noise mode. Here the overall decreased tendencies of SSMVEP amplitude and accuracy in non-noise tasks and their stable tendencies in noise-tagged tasks could be noticed across consecutive on-task trials in Figure 6.

Overall, the normalized response traces for the noise-tagged BCI task remained stable regarding amplitude, SNR, and accuracy over the range of successive trials at both stimulation frequencies (i.e., 15 Hz and 8.57 Hz). There was no significant linear (*p* > 0.05 for 15 Hz and 8. 57 Hz) or quadratic (*p* > 0.05 for 15 Hz and 8.57 Hz) association between the trial order and amplitude. Similar results were found for SNR and accuracy at both stimulation frequencies (ANOVA testing for linear trend: *p* > 0.05 for all comparisons; ANOVA testing for quadratic trend: *p* > 0.05 for all comparisons). The SSMVEP amplitude, SNR and accuracy differences were analyzed with one-way ANOVA, and the significance level was adjusted by means of Bonferroni correction controlling for all pairwise comparisons of the successive trials. Here, one-way ANOVA with Bonferroni-corrected post-hoc tests also revealed no significant fatigue effect in amplitude (*p* > 0.05 for 15 Hz and 8.57 Hz), SNR (*p* > 0.05 for 15 Hz and 8.57 Hz) and accuracy (*p* > 0.05 for 15 Hz and 8.57 Hz) between different trial orders within the noise-tagged task. Furthermore, the overall amplitude and SNR performance under the noise-tagged condition was significantly more superior beyond the non-noise condition at 15 Hz (one-way ANOVA: *p* < 0.001 for all comparisons). These indicated that visual noise could facilitate the alleviation of mental fatigue during the noise-tagged BCI task.

For the non-noise task, the performance at 15 Hz still presented a roughly stable tendency regarding amplitude, SNR and corresponding online accuracy across successive trials. No significant SSMVEP amplitude, SNR and accuracy differences were found between different trial orders (*F*(4, 270) = 1.63, *p* = 0.167 for amplitude; *F*(4, 270) = 0.96, *p* = 0.430 for SNR; *F*(4, 55) = 1.25, *p* = 0.299 for accuracy; one-way ANOVA with Bonferroni-corrected post-hoc tests). Exceptions were found at 8.57 Hz as dramatically-significant decreasing trends in amplitude (ANOVA testing for linear trend: *p* < 0.001), SNR (*p* = 0.004), and accuracy (*p* = 0.005) were observed as time elapsed. The SSMVEP amplitude, SNR and accuracy differences between different trial orders were also significant (*F*(4, 270) = 4.78, *p* < 0.001 for amplitude; *F*(4, 270) = 3.43, *p* = 0.009 for SNR; *F*(4, 270) = 3.15, *p* = 0.021 for accuracy; one-way ANOVA with Bonferroni-corrected post-hoc tests), i.e., the lowest amplitude, SNR, and accuracy were associated with the highest trial order. All these findings demonstrated that the noise-tagged paradigm exhibited a superior anti-fatigue efficacy and even better performance than the conventional non-noise paradigm during prolonged BCI usage.

**Figure 7:**
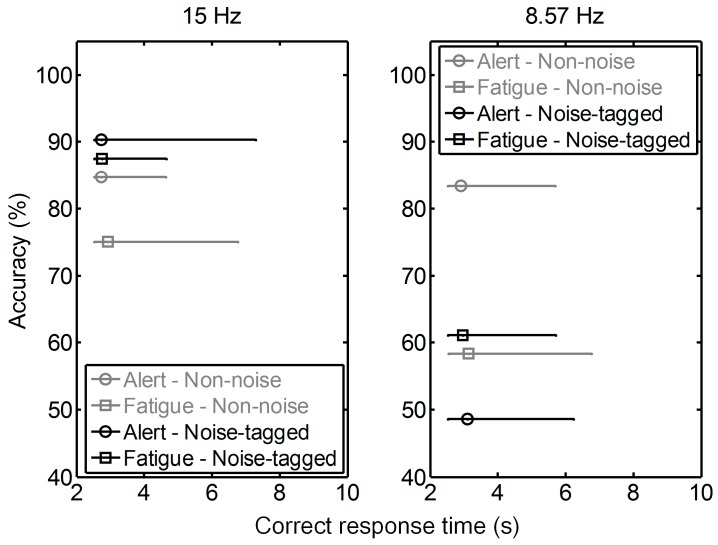
Online accuracy and correct response time in the alert versus fatigue state in both non-noise and noise-tagged BCI tasks across participants. The grand-averaged online accuracy and corresponding correct response time were calculated in semi-synchronous online BCI tasks performed across twelve participants. Results are shown in black for the noise-tagged task and in gray for the non-noise task. Each error bar characterizes the distribution of correct response time upon a certain online accuracy value, which was calculated as the grand-averaged percentage of correctly-judged trials. The upper and lower bounds of each error bar were set with maxima and minima of the time distribution, and the central point (circle and square) represents the mean. For convenience, the upper end of the ordinate was set above 100%.

To support the above-mentioned accuracy results in Figure 6, more sophisticated statistical analysis with correct response time, which characterized BCI efficiency, were assessed under different noise strengths, as illustrated in Figure 7. The differences in correct response time upon a certain online accuracy value for both alert and fatigue states were analyzed in the non-noise and noise-tagged BCI tasks across twelve participants. This accuracy value was calculated as the grand-averaged percentage of correctly judged trials, and the correct response time at stimulation frequencies of 15 Hz and 8.57 Hz in the first and last single trials of all experimental runs was extracted to represent the alert and fatigue states, respectively. 

After the 15-Hz stimulation, both accuracy and efficiency prevailed in the alert state rather than in the fatigue state in the normal non-noise task. Online accuracies decreased by 11% from the alert state to the fatigue state; however, this effect did not reach statistical significance (one-way ANOVA: *F*(1, 22) = 2.93, *p* = 0.101). A less significant 8% increment in the requirement of correct response time was also found in the fatigue state as compared to the alert state in the non-noise task (alert state: 2.72 ± 0.46 s vs. fatigue state: 2.93 ± 0.92 s; one-way ANOVA: *F*(1, 108) = 2.39, *p* = 0.125), and a significantly larger variation in the correct response time could be observed in the fatigue state (two-sample *F*-test: *p* < 0.001). The results indicated that the brain slowed its activity due to the reduced cognitive capacity during fatigue. For the noise-tagged task, the online accuracy and correct response time between the alert and fatigue states seemed to exhibit comparable performance (*F*(1, 22) = 0.01, *p* = 0.908 for accuracy; alert state: 2.74 ± 0.75 s vs. fatigue state: 2.75 ± 0.48 s, one-way ANOVA: *F*(1, 108) = 0.01, *p* = 0.905 for correct response time). A significantly smaller variation of the correct response time was observed in the fatigue state as compared to the alert state (two-sample *F*-test: *p* < 0.001).

A similar phenomenon occurred at 8.57 Hz: the fatigue state resulted in comparable online accuracy and correct response time to the alert state in the noise-tagged task (*F*(1, 22) = 0.57, *p* = 0.458 for accuracy; alert state: 3.09 ± 0.93 s vs. fatigue state: 2.96 ± 0.71 s, one-way ANOVA: *F*(1, 108) = 0.51, *p* = 0.475 for correct response time). The non-noise task still presented higher accuracy during the alert state than during the fatigue state. The online accuracies dramatically decreased by 30% from the alert state to the fatigue state (one-way ANOVA: *F*(1, 22) = 15.92, *p* < 0.001), whereas the changes in the correct response time between the alert and fatigue states did not reach statistical significance (one-way ANOVA: *F*(1, 108) = 1.87, *p* = 0.175).

The above online accuracy results refer to each target being correctly detected corresponding to the cue on the screen, i.e., the true positive rate (TPR). A more sophisticated analysis of the false positive rate (FPR, i.e., a target was detected, but it was different from the cue on the screen) was conducted in addition to the TPR analysis. The FPR obtained at 15 Hz indicated the percentage of trials judged as 15 Hz when 8.57-Hz or 12-Hz cues were displayed on the screen. FPR at 8.57 Hz indicated the percentage of trials judged as 8.57 Hz when 12-Hz or 15-Hz cues were displayed on the screen. The TPR and FPR results of individual participants are presented in Table 1. One-way ANOVA revealed that almost all participants exhibited TPR improvement in the noise-tagged task at 15 Hz except for Participant 3. A comparable, or even reversed, phenomenon could be observed at 8.57 Hz, which may be due to the low-pass property of the sensory systems highlighted in our previous study [23] and to the fact that the visual noise strength adopted in the present study wielded little beneficial influence on this specific frequency. More interestingly, three-way repeated ANOVA with Bonferroni correction, which included the factors of “noise mode”, “stimulation frequency”, and “accuracy mode” using TPR vs. FPR conditions, showed a significant FPR difference between non-noise and noise-tagged tasks (*p* < 0.001). Specifically, the FPRs significantly decreased during the application of the visual noise task as compared to the non-noise task at 8.57 Hz (noise-tagged: 8.72% ± 8.33 vs. non-noise: 35.04% ± 17.25, one-way ANOVA: *F*(1, 22) = 22.65, *p* < 0.001). The same trend also existed at 15 Hz (noise-tagged: 16.76% ± 8.42 vs. non-noise: 31.74% ± 8.75, one-way ANOVA: *F*(1, 22) = 18.24, *p* < 0.001), but to a significant smaller extent (one-way ANOVA: *F*(1, 22) = 5.53, *p* = 0.028). This may be due to the fact that the performance obtained at 15 Hz benefitted largely from the visual noise which, in turn, resulted in the FPR decrement at 8.57 Hz. Similarly, the small performance improvement observed during the noise-tagged task at 8.57 Hz brought less beneficial influence on the FPR decrement at 15 Hz.

**Figure 8:**
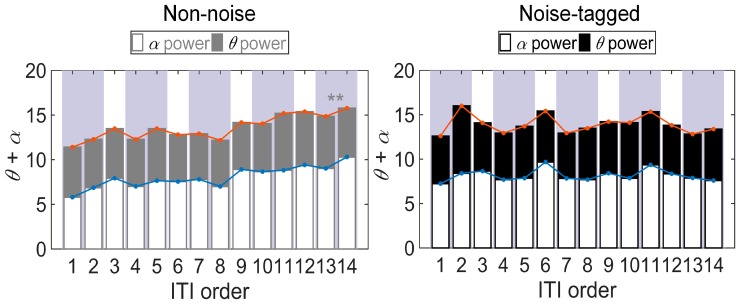
Stacked histograms showing the comparison of different fatigue indices (i.e., *α*, *θ*, and *θ* + *α* powers) between 14 consecutive non-task ITIs in both non-noise and noise-tagged conditions across participants. The grand-averaged mean values and SD of *α*, *θ*, and *θ* + *α* powers were calculated from 14 consecutive non-task ITIs of the same order in each run across twelve participants. The polyline in blue represented the time course of the grand-averaged *α* power along with the 14 ITIs within five stimulation sequences of multiple runs. The polyline in magenta represented the time course of the grand-averaged *θ* + *α* power. All statistics were assessed by one-way ANOVA, ** *p* < 0.01 between 14 consecutive ITIs.

In addition to the absolute amplitude, SNR, and accuracy, the EEG power indices (i.e., *α*, *θ,* and *θ* + *α*) within ITIs might provide information about the fatigue-like modulation of visual stimulation. The increasing literature on this topic showed that the resting brain functions are linked to individual differences in cognitive function and behavioral performance, including visual cognition and attention, memory recall, language processing, and decision-making [26]. Moreover, resting spontaneous EEGs have been observed in response to changes in mental fatigue [12]. With the same paradigms, but by examining the spontaneous oscillations rather than on-task EEGs, the stacked histograms presented in Figure 8 revealed how the grand-averaged *α*, *θ*, and *θ* + *α* powers evolved along the 14 ITIs within five stimulation sequences in multiple runs of twelve participants. In each panel, the two ITIs enclosed in each subgroup in purple represented the intervals within one stimulation sequence. 

Across all participants, consistent tendencies were obtained in *α* and *θ* + *α* powers as these two indices increased positively and significantly from the start to the end of the stimulation in the non-noise task (ANOVA testing for linear trend: *p* < 0.001 for all comparisons). However, there was no significant correlation trend between the ITI order and *θ* power in the non-noise task (ANOVA testing for linear trend: *p* = 0.392), as well as between ITI order and *α*, *θ*, and *θ* + *α* powers in the noise-tagged task (ANOVA testing for linear trend: *p* > 0.05 for all comparisons). The *α*, *θ*, and *θ + α* power differences were analyzed with one-way ANOVA, and the significance level was adjusted by means of Bonferroni correction controlling for all pairwise comparisons of the successive ITI subgroups. Here one-way ANOVA with Bonferroni-corrected post-hoc tests revealed an expected increase of mental fatigue with a significantly larger *α* power in the fifth ITI subgroup than in the first ITI subgroup occurred within the non-noise task (*F*(4, 655) = 4.21, *p* = 0.002). The subsequent *θ + α* power was also significant (*F*(4, 655) = 3.52, *p* = 0.007), whereas the fatigue effect on *θ* power was non-significant (*F*(4, 655) = 0.69, *p* = 0.601). For the noise-tagged task, no significant fatigue effect could be observed (*F*(4, 655) = 0.56, *p* = 0.693 for *α* power; *F*(4, 655) = 1.36, *p* = 0.246 for *θ* power; *F*(4, 655) = 0.95, *p* = 0.436 for *θ + α* power; one-way ANOVA with Bonferroni-corrected post-hoc tests).

## 4. Discussion

Considering the previous studies on mental load, a high mental load may result in a decrement in the user's arousal level and in task performance. In addition, increases in task difficulty and in mental load may lead to drowsiness which may, in turn, increase the mental effort necessary to stay alert and, thus, cause fatigue. Fatigued people often experience difficulties in focusing their attention and appear more easily distractible. In this work, we showed that presenting a moderate spatiotemporal visual noise to participants can reliably alleviate the mental load and the level of fatigue during online operation of a periodic visual stimulation-based BCI task that places demands on the attentional processes. This implies that the human visual system may exploit the power of randomness to enhance higher brain functions, such as the visual attention control. Two elaborate analyses were implemented in this study. First, we did observe a decreased *α* band power with the increment in cognitive load during the non-noise BCI task. We also reported an expected rise in *θ* power with the increase in mental load, although its effect seemed to be present, but less clear. The reason why we reported an obvious *α* power change rather than a *θ* power change may be due to the fact that *θ* waves often occur in the frontal and/or fronto-central sites, whereas *α* rhythms were dominated by the parietal and/or parieto-occipital areas [23], which is the exact place we recorded the SSMVEP signals. Second, the SSMVEP amplitude, SNR, and online accuracy significantly declined as affected by participants’ fatigue in the non-noise BCI task. Additionally, a much larger variation of the correct response time could be observed in the post-viewing as compared to the initial viewing in the non-noise task. Furthermore, the number of missed targets also increased significantly during the non-noise task. The rather dramatic increase in missed targets was not only due to a simple reduction in the number of correct responses: the number of responses to non-targets even increased. This suggested that the observed performance deterioration in the non-noise BCI task was not only caused by a task disengagement, but may also derive from a reduction in goal-directed attention, leaving participants performing in a more stimulus-driven fashion. To a large extent, this effect seems to be caused by an inability of fatigued participants to efficiently recruit and allocate their attentional resources [53]. However, for the noise-tagged BCI task, the SSMVEP amplitude, SNR, and online accuracy benefited from the addition of visual noise and did not show any significant changes during prolonged BCI usage. Furthermore, in the noise-tagged condition, the *α* and *θ* + *α* powers of resting spontaneous EEGs exhibited roughly stable tendencies, whereas tendencies toward increased *α* and *θ* + *α* powers could be observed in the non-noise condition with the increase in ITI order. These suggested that with the modulation of attention by noise, there may exist less demands of continuous attention toward specific targets when performing the visual attention task, leading to the compensation of performance deterioration that occurs in the conventional stimulation paradigm.

Stochastic resonance (SR), or stochastic facilitation, occurs in a wide range of non-living and living systems. Several studies have shown an importantly beneficial role of noise in information processing at a neuronal level and in the primary sensory systems of animals. In humans, SR has been demonstrated in peripheral organs and in tactile, hearing, and vision processing within the brain [54,55,56,57,58,59,60]. Additionally, SR has even been found in higher cognitive functions (e.g., visual attention and arousal level controlling) which would involve widely separated brain regions above the “low-level” sensory systems [61]. For the visual perception, the dynamic interaction between noise and visual signals in the human brain may make noise mediate the visual selective attention and arousal by which the activities in lower- and higher-level visual and association areas are temporarily coordinated [27,62]. In 2003, Kitajo et al. [63] suggested that the added noise enhances phase synchronization both within the visual areas of the brain where the noise and the signal representation are combined, and between the visual areas and other brain areas responsible for generating visual attention control. Here, noise plays a role as an integral part of inter-neuronal communication and less than optimal amounts have less of an effect, and larger than optimal amounts dissolve synchronization [64,65,66]. Regarding the effects of noise on synchronization, noise, in a general way, increases arousal, which makes the participants more alert, and less drowsy.

In addition to the role of noise in neural synchronization, which establishes transient networks that implement perceptual and cognitive processes, such as memory, attention, and even conscious awareness [35,67], the multi-stable nature of visual attention would let noise influence the switching among different attention states in a visual scene. Visual attention is a dynamic multi-stable process where the attention “spotlight” typically shifts from one spatial location or target in a visual scene to another one over a period of less than a second to a few seconds, either voluntarily or passively, or exclusively or intermittently [68]. Thus, we can regard the movement of the focus of attention as the movement among stable points in a visual scene. In this case, each stable point would represent a particular location or target. Due to this multi-stable nature of visual attention, noise at the cognitive level is associated with enhanced switching behavior among attention states [69]. Thus, with optimal noise strength, the hopping among the multiple stable points should be stochastically synchronized with the input signals and the visual attention ability should be improved.

## 5. Conclusions

Taken together, our work suggests that participants developed increasing difficulties in staying alert and sustaining attention in the conventional non-noise paradigm. However, they could continue to successfully accomplish the BCI usage at an acceptable mental load and fatigue level with the addition of visual noise. This finding will help us to understand how the noise-facilitated attention control alleviates mental load and fatigue. In addition to EEG characteristics that were used in the evaluation of mental load and fatigue, other measures can be considered to provide additional information for monitoring the mental states more efficiently in future directions of the current study, e.g., incorporating other monitoring techniques to construct a hybrid BCI system such as hybrid fNIRS-EEG BCI [70,71] and BCI/eye-tracker systems [11]. Moreover, more noise strengths and larger frequency ranges with decreased frequency differences could be involved in future work for further sophisticated studies of SR fingerprinting in arousal-mediated BCI applications.

## Figures and Tables

**Table 1:** The TPR and FPR results of individual participants.

Participants	15 Hz				8.57 Hz			
	Non-Noise		Noise-Tagged		Non-Noise		Noise-Tagged	
	TPR (%)	FPR (%)	TPR (%)	FPR (%)	TPR (%)	FPR (%)	TPR (%)	FPR (%)
1	95	40	100	0	88	36	56	0
2	90	15	95	5	85	35	92.5	2.5
3	90	35	85	25	65	20	70	5
4	90	40	96.67	13.33	56.67	23.33	66.67	3.33
5	86.67	20	93.33	13.33	70	20	50	15
6	76.67	33.33	86.67	23.33	84	52	68	8
7	53.33	26.67	80	20	53.33	46.67	80	20
8	80	36	96	20	70	20	100	0
9	73.33	43.33	100	16.67	66.67	26.67	56.67	6.67
10	60	30	100	25	35	40	65	10
11	62.5	37.5	80	27.5	62.5	77.5	60	27.5
12	60	24	72	12	80	23.33	53.33	6.67
